# Dietary Knowledge and Its Associated Factors Among Patients with Diabetes and Hypertension in Tertiary Hospitals in Dodoma, Tanzania: A Cross-sectional Study

**DOI:** 10.24248/eahrj.v9i2.850

**Published:** 2025-12-24

**Authors:** Elizabeth Madikenya, Erick Donard Oguma, Osward Sevin Lyimo, Julius E. Ntwenya, Leornard Katalambula, Stephen M. Kibusi

**Affiliations:** a Department of Clinical Nursing, School of Nursing and Public Health, The University of Dodoma, Benjamin Mkapa Road, Iyumbu, Dodoma, Tanzania; b Department of Nursing Management and Education, School of Nursing and Public Health, The University of Dodoma, Benjamin Mkapa Road, Iyumbu, Dodoma, Tanzania; c Department of Public Health and Community Nursing, School of Nursing and Public Health, The University of Dodoma, Benjamin Mkapa Road, Iyumbu, Dodoma, Tanzania

## Abstract

**Background:**

Dietary knowledge plays a central role in the prevention and management of non-communicable diseases (NCDs) such as diabetes and hypertension. However, limited evidence exists on patients’ understanding of appropriate dietary practices within the Tanzanian context. This study assessed the level of dietary knowledge and its associated factors among patients attending two tertiary hospitals in Dodoma, Central Tanzania.

**Methods:**

An analytical cross-sectional study was conducted among adults diagnosed with diabetes, hypertension, or both at Benjamin Mkapa Hospital and Dodoma Regional Referral Hospital between July and September 2021. A total of 335 participants were selected through simple random sampling. Data was collected using a structured interviewer-administered questionnaire, and dietary knowledge was categorized as adequate or inadequate based on the mean score. Multivariate logistic regression was performed to identify factors associated with inadequate dietary knowledge, with significance set at *p*<.05.

**Results:**

Nearly half of the respondents, 47.8% (n = 160) had hypertension, 21.2% (n = 71) had diabetes, and 31.0% (n = 104) had both conditions. More than half, 53.4% (n = 179), demonstrated inadequate dietary knowledge. Middle-aged adults (30–44 years) (AOR = 3.89; 95% CI: 1.76–8.61), unemployed individuals (AOR = 2.62; 95% CI: 1.36–5.05), and those who consumed alcohol (AOR = 2.20; 95% CI: 1.18–4.12) had significantly higher odds of inadequate dietary knowledge. Conversely, participants with hypertension only had lower odds of inadequate knowledge compared with those with both diabetes and hypertension (AOR = 0.42; 95% CI: 0.23–0.77).

**Conclusion:**

This study revealed that more than half of patients living with diabetes, hypertension, or both had inadequate dietary knowledge, highlighting a significant gap in the nutritional understanding required for effective NCD self-management. Hence, underscore the need for targeted, context-specific dietary education interventions, particularly for socio-economically vulnerable groups, to strengthen NCD management and improve long-term health outcomes in Tanzania.

## BACKGROUND

Non-Communicable Diseases (NCDs) remain the leading global cause of mortality, accounting for approximately 74% of all deaths, an estimated 41 million deaths annually.^1^ Of these, premature deaths before age 70 affect about 28 individuals every minute, with 86% occurring in Low- and Middle-Income Countries (LMICs). Cardiovascular diseases, cancers, chronic respiratory diseases, and diabetes collectively contribute nearly 80% of all premature NCD deaths, underscoring the growing global health crisis.^2^ Over the past two decades, sub-Saharan Africa has experienced a marked rise in NCD prevalence, driven largely by modifiable lifestyle factors including unhealthy diets, physical inactivity, tobacco use, harmful alcohol consumption, and growing rates of obesity.^3,4^ Recognizing this burden, the United Nations Sustainable Development Goals (SDG 3.4) call for a one-third reduction in premature NCD mortality by 2030.

Diabetes mellitus and hypertension are among the most common NCDs globally and remain major contributors to morbidity and mortality. Diabetes currently affects 537 million people worldwide, most of whom reside in LMICs, and was responsible for an estimated 6.7 million deaths in 2021.^5^ Hypertension affects nearly 1.3 billion adults aged 30 to 79 years, yet only 54% are diagnosed, 42% receive treatment, and 21% have controlled blood pressure.^6^ Effective management of both conditions rely heavily on dietary modification and regular physical activity, which are central to delaying complications and improving long-term outcomes.

According to the 2019 WHO Hypertension Profile for Tanzania, approximately 4.9 million adults (30–79 years) are living with hypertension, yet only 34% are diagnosed, 15% receive treatment, and only 7% achieve adequate blood pressure control.^7^ Achieving the global target of 50% control would require effective treatment of an additional 2.1 million Tanzanians, a gap far larger than that observed in neighbouring countries such as Rwanda, Burundi, and Uganda. These figures highlight the urgent need to strengthen hypertension prevention and management strategies, including improved access to dietary education.

Diet is one of the most influential modifiable risk factors for NCDs,^8,9^ with strong evidence linking unhealthy dietary patterns such as high intake of sugar, salt, saturated fats, and refined carbohydrates to increased risk of diabetes, hypertension, obesity, and cardiovascular diseases.^10–13^ Conversely, diets rich in fruits, vegetables, whole grains, and low-fat foods significantly reduce disease risk. Despite this evidence, many individuals “consume food but not diet,” meaning they lack the foundational knowledge of what to eat, in what quantities, and how dietary composition influences disease prevention and management.^14–24^

Although diabetes and hypertension continue to rise in Tanzania, little is known about the level of dietary knowledge and its associated factors among patients living with these conditions, despite diet being a cornerstone of effective NCD management. Understanding these factors is critical, as inadequate dietary knowledge undermines self-management, contributes to poor disease control, and increases the burden of complications. Addressing these gaps is therefore essential to improving clinical outcomes and strengthening NCD care in Tanzania. This study therefore aims to assess the level of dietary knowledge and its associated factors among patients with diabetes and hypertension attending two tertiary referral hospitals in Dodoma, Central Tanzania.

## METHODS

### Study Design and Setting

This was an analytical cross-sectional, hospital-based study conducted at Benjamin Mkapa Hospital (BMH) and Dodoma Regional Referral Hospital (DRRH), both located in Dodoma Region, Tanzania. Data was collected between July and September 2021. These two tertiarylevel facilities serve as major referral centres for chronic disease management in the central zone, attracting a diverse population of patients with varying socio-demographic and clinical backgrounds.

### Study Population

The study population comprised adult patients diagnosed with diabetes mellitus, hypertension, or both, who were receiving routine outpatient care at Benjamin Mkapa Hospital (BMH) and Dodoma Regional Referral Hospital (DRRH) in Dodoma Region, Tanzania.

### Eligibility Criteria

Participants were eligible for inclusion if they were adults aged 18 years or older with a confirmed diagnosis of diabetes mellitus, hypertension, or both, and were attending follow-up care at the medical outpatient clinics of Benjamin Mkapa Hospital (BMH) or Dodoma Regional Referral Hospital (DRRH) during the study period. Only patients who were clinically stable, able to communicate, and willing to provide written informed consent were enrolled, ensuring reliable and accurate responses to the interview-based questionnaire. Patients were excluded if they were critically ill, exhibited cognitive or communication impairments, or were otherwise unable to meaningfully participate at the time of data collection.

### Sample Size Estimation

The sample size was determined using Cochran's formula for a single-population proportion, using a 60% prevalence of adequate dietary knowledge, derived from a comparable study conducted in Uganda, an East African country with similar socio-economic and health system characteristics.^25^ Using a 95% confidence level (Z = 1.96), 5% margin of error, and the formula N = Z^2^ P(1–P)/E^2^, the minimum required sample size was 369 participants. Of the 369 eligible participants approached, 335 consented and completed the study, yielding a response rate of 90.8%.

### Sampling Technique

A simple random sampling technique was utilized to select participants from a sampling frame of all eligible patients attending diabetes and hypertension outpatient clinics at BMH and DRRH during the study period. Each clinic prepared a daily list of patients who met the inclusion criteria, forming the sampling frame for that day. Using this list, participants were randomly selected through a computer-generated random number method to ensure that every eligible patient had an equal and independent chance of being included in the study. This approach minimised selection bias and strengthened the representativeness of the sample within the clinic population.

### Data Collection Method

Data was collected through face-to-face interviewer-administered interviews using a structured questionnaire. Trained research assistants conducted the interviews in designated clinic areas to ensure privacy and comfort for the participants. Before data collection, the assistants received training on study procedures, interview techniques, ethical considerations, and standardization of questionnaire administration to minimize interviewer bias. Eligible patients were approached after receiving their clinical services, the study purpose was explained, and written informed consent was obtained. Each interview session lasted approximately 15 to 20 minutes. This approach ensured high response accuracy, accommodated varying literacy levels among participants, and supported consistent and reliable data collection across both study sites.

### Data Collection Tool

Data was collected using a structured questionnaire that was adapted from a previously validated tool developed in Limpopo, South Africa.^26^ The questionnaire consisted of two sections: (1) socio-demographic and clinical characteristics (11 items), and (2) dietary knowledge assessment (10 items) measured on a three-point Likert scale (“Yes,” “Not sure,” “No”). The tool was initially prepared in English and subsequently translated into Swahili, followed by back-translation to ensure linguistic accuracy and conceptual equivalence. Content validity was reviewed by experts in public health, nutrition, and epidemiology. A pilot study involving 10% of the anticipated sample was conducted at a non-study facility to assess clarity, relevance, and reliability of the instrument. Based on pilot results, minor adjustments were made, and the dietary knowledge scale demonstrated acceptable internal consistency with a Cronbach's alpha of 0.78. This ensured that the tool was valid, culturally appropriate, and reliable for use among Tanzanian NCD patients.

### Variables Description and Measurement

#### Independent Variables

The independent variables in this study comprised a range of socio-demographic and clinical characteristics that were hypothesized to influence dietary knowledge. These included; participants’ age, sex, marital status (single, married, divorced/separated, or widowed), education level (no formal education, primary, secondary, or tertiary), occupation (employed, unemployed, self-employed, farmer, or other categories), and religion. Clinical characteristics included family history of NCDs (yes/no) and type of diagnosis, classified as diabetes mellitus, hypertension, or both conditions. These variables were selected based on existing evidence showing their potential association with knowledge-related health outcomes, and each was coded appropriately for analysis in the logistic regression model. The multivariate logistic regression analysis was used to find the association between these factors with the outcome variable (dietary knowledge), at a significance level of 0.05 (95% CI).

#### Dependent Variable

The primary outcome variable was dietary knowledge. Dietary knowledge was assessed using 10 questions measured on a three-point scale (“Yes,” “Not sure,” “No”), scored as 2, 1, and 0 respectively. Individual item scores were summed to generate a total dietary knowledge score for each participant. The mean score was used as the cut-off point, with participants scoring ≥ mean classified as having adequate dietary knowledge, and those scoring < mean classified as having inadequate dietary knowledge. The use of the mean as a cut-off was justified by the absence of an established standard threshold for the adapted tool within the Tanzanian context.

### Data Analysis

Data was entered and analysed using SPSS version 26. Prior to analysis, the dataset was checked for completeness, cleaned, and assessed for normality. Descriptive statistics, including frequencies, percentages, means, and standard deviations, were used to summarize socio-demographic characteristics and dietary knowledge scores. Bivariate analysis was conducted to explore the relationship between dietary knowledge (dependent variable) and each independent variable. Variables with a *p-*value < .20 in the bivariate analysis were included in a multivariate logistic regression model to identify factors independently associated with dietary knowledge. Adjusted Odds Ratios (AORs) with 95% Confidence Intervals and corresponding *p-*values <.05 were used to determine statistical significance.

### Ethical Consideration

The study received an ethical approval from the University of Dodoma Institutional Research Review Committee with (Ref. No, MA.84/261/01/). Additional permissions to conduct the study were granted by the Dodoma Municipal Director, the District Medical Officer, and the administrative authorities of BMH and DRRH. Prior to participation, all eligible respondents were provided with detailed information about the study's objectives, procedures, potential risks, and benefits. Written informed consent was obtained from each participant before the interview. Confidentiality was strictly maintained by using anonymous codes instead of personal identifiers, and all collected data were securely stored and accessed only by the research team. Participation was entirely voluntary, and respondents were informed of their right to withdraw at any point without any consequences for their clinical care.

## RESULTS

### Socio-demographics Characteristics of Study Participants (N=335)

A total of 335 patients with diabetes and hypertension were enrolled in this study, of whom 127 (37.9%) were male and 208 (62.1%) were female. The mean age of respondents was 50 years (SD = 15.5), with ages ranging from 20 to 85 years. Older adults (≥ 60 years) constituted the largest age group, representing 108 (32.2%) of participants. More than half of the respondents, 182 (54.3%), resided in rural areas. The majority were married, accounting for 235 (70.1%), and most had completed primary education 206 (61.5%). A large proportion of participants identified as Christian (58.2%) and were self-employed (58.5%). Monthly income varied widely, with some participants reporting no earnings and others earning up to 940,000 TSH, while the mean monthly income was 102,134.33 TSH. Overall, most participants (60.6%) had low monthly income.

Moreover, nearly half of the respondents, 47.8% (n = 160), had hypertension, 21.2% (n = 71) had diabetes, while 31.0% (n = 104) were living with both conditions. With regard to the duration of illness, approximately 75% (n = 251) had been diagnosed more than five years ago. In addition, most respondents reported having no family history of diabetes or hypertension (Table 1).

**TABLE 1:** Socio-Demographics Characteristics of Study Participants (N=335)

Variables	Frequency (n)	Percentage (%)
Age groups		
18–29 years (Young adults)	71	21.2
30–44 years (Middle adults)	97	29.0
45–59 years (Adults)	59	17.6
≥ 60 years (Older adults)	108	32.2
Gender		
Male	127	37.9
Female	208	62.1
Area of residence		
Urban area	153	45.7
Rural area	182	54.3
Marital status		
Single	32	9.6
Married	235	70.1
Divorced	12	3.6
Widow/widower	56	16.7
Education level		
Primary education	206	61.5
Secondary education	91	27.2
College/University	15	4.5
Never being in school	23	6.9
Religion		
Christian	195	58.2
Muslim	140	41.8
Occupation status		
Unemployed	80	23.9
Employed	59	17.6
Self employed	196	58.5
Monthly average income		
Very Low income	203	60.6
Low income	75	22.4
Moderate income	22	6.6
High income	35	10.4
Chronic disease suffering from		
Hypertension	160	47.8
Diabetes	71	21.2
Both diabetes and hypertension	104	31.0
Duration of disease condition		
1 month–11 months	92	27.5
1 year–5 years	159	47.5
6 years–10 years	65	19.4
Above 10 years	19	5.7
Family history of diabetes or hypertension		
Yes	119	35.5
No	216	64.5

### Dietary Knowledge among Diabetic and Hypertensive Patients

**FIGURE 1:**
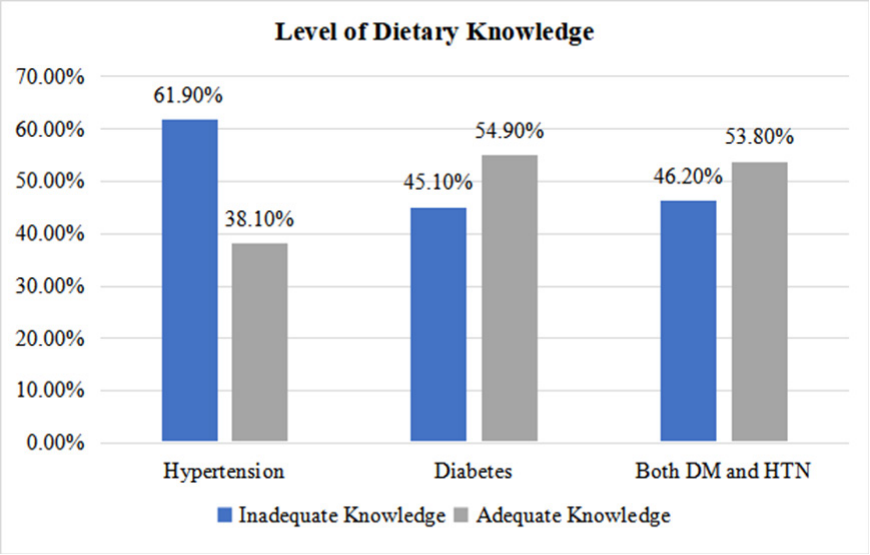
The Proportion of Dietary Knowledge among Patients with Diabetes and Hypertension.

More than half of the respondents, 53.4% (n = 179), demonstrated inadequate dietary knowledge. When comparing disease categories, a large proportion of hypertensive patients (61.9%) had inadequate dietary knowledge compared with those diagnosed with diabetes (Figure 1).

The majority of participants, 71.9% (n = 241), were not aware that consuming highly processed and junk foods contributes to the development of NCDs. Additionally, about 81% (n = 271) of the participants were unsure whether dietary management is essential for individuals with diabetes and hypertension. More than half of the respondents, 51.3% (n = 172), lacked awareness that whole grains, fruits, and vegetables are important components of meal planning for patients with type 2 diabetes and hypertension. Furthermore, approximately 27% (n = 90) were uncertain whether consuming plant-based oils, such as olive oil, poses a lower risk for hypertension and diabetes compared with diets high in sugar, salt, animal fats, and fatty foods (Table 2).

**TABLE 2:** Respondents’ Responses Regarding Dietary Knowledge (N=335)

Items	Frequency	Percentage
Non communicable diseases NCDs refer to the chronic diseases that cannot be transmitted from one person to another.		
No	0	0.0%
Not sure	189	56.4%
Yes	146	43.6%
Diabetes and Hypertension are examples of non-communicable diseases		
No	0	0.0%
Not sure	127	37.9%
Yes	208	62.1%
Improper lifestyle like poor dietary modification is the major leading cause of non-communicable diseases		
No	81	24.2%
Not sure	118	35.2%
Yes	136	40.6%
Meal planning (nutrition management) is essential in diabetic and hypertensive patients		
No	0	0.0%
Not sure	271	80.9%
Yes	64	19.1%
Eating highly processed foods and junk foods contribute to development of non-communicable diseases		
No	241	71.9%
Not sure	81	24.2%
Yes	13	3.9%
High quantity of oils from plants such as olive has low risk of hypertension and diabetes than taking high amounts of sugary, salty, animal fat and fatty foods		
No	32	9.6%
Not sure	90	26.9%
Yes	213	63.6%
Fats and sugars should be taken in small amounts so as to maintain healthy life and prevent acquiring of non-communicable diseases		
No	100	29.9%
Not sure	13	3.9%
Yes	222	66.3%
Unhealthy diet and poor exercise habits contribute to increased chance of acquiring non-communicable diseases		
No	0	0.0%
Not sure	138	41.2%
Yes	197	58.8%
Complex carbohydrates such as whole grains, fruits and vegetables are good for meal planning for individuals with diabetes especially type 2? or hypertension		
No	172	51.3%
Not sure	137	40.9%
Yes	26	7.8%
We should plan daily our meals so as to maintain a good health and be at a reduced risk of developing non communicable diseases		
No	51	15.29%
Not sure	5	1.5%
Yes	278	83%

### Factors Associated with Dietary Knowledge among Patients with Diabetes and Hypertension

Multivariate logistic regression revealed several factors independently associated with dietary knowledge among patients with chronic conditions. Middle-aged adults (30–44 years) demonstrated significantly higher odds of having inadequate dietary knowledge compared with older adults (≥ 60years) (AOR = 3.89; 95% CI: 1.76–8.61; *p* = .001). Unemployed participants also had significantly greater odds of inadequate knowledge compared with those who were self-employed (AOR = 2.62; 95% CI: 1.36–5.05; *p* = .004). Furthermore, individuals diagnosed with hypertension only were significantly less likely to have inadequate dietary knowledge than those with both hypertension and diabetes (AOR = 0.42; 95% CI: 0.23–0.77; *p* =.005). In addition, participants who consumed alcohol had higher odds of inadequate dietary knowledge compared with non-drinkers (AOR = 2.20; 95% CI: 1.18–4.11; *p*=.013). No other socio-demographic or clinical factors showed statistically significant associations with dietary knowledge in the adjusted model (Table 3).

**TABLE 3:** The Multivariate Binary Logistic Regression Analysis Showing Factors Associated with Dietary Knowledge Among Patients with Diabetes and Hypertension (N=335)

Variable	Knowledge Status		AOR	95% CI		p-value
	Inadequate	Adequate		Lower	Upper	
Age groups						
18–29 years (Young adults)	43	28	1.02	0.38	2.78	.965
30–44 years (Middle adults)	37	60	3.89	1.76	8.61	.001
45–59 years (Adults)	34	25	1.09	0.49	2.45	.834
≥ 60 years (Older adults)	65	43	Ref	-	-	-
Gender						
Male	57	70	1.67	0.97	2.94	.064
Female	122	86	Ref	-	-	-
Area of residence						
Urban area	81	72	0.90	0.54	1.50	.682
Rural area	98	84	Ref	-	-	-
Marital status						
Single	16	16	0.90	0.25	3.26	.874
Married	128	107	0.67	0.32	1.43	.300
Divorced	8	4	0.33	0.08	1.44	.141
Widow	27	29	Ref	-	-	-
Education level						
Primary education	123	83	0.52	0.19	1.39	.190
Secondary education	36	55	1.71	0.60	4.92	.316
College/University	9	6	0.37	0.07	2.13	.267
Never being in school	11	12	Ref	-	-	-
Religion						
Christian	106	89	1.10	0.63	1.89	.745
Muslim	73	67	Ref	-	-	-
Occupation status						
Unemployed	39	41	2.62	1.36	5.05	.004
Employed	26	33	2.08	0.85	5.08	.108
Self employed	114	82	Ref	-	-	-
Reported average monthly income						
Low income	162	139	1.27	0.29	5.44	.752
Moderate income	9	10	1.23	0.24	6.40	.804
High income	8	7	Ref	-	-	-
Type of chronic disease						
Hypertension	99	61	0.42	0.23	0.77	.005
Diabetes	32	39	1.13	0.54	2.36	.739
Both diabetes and hypertension	48	56	Ref	-	-	-
Duration of disease condition						
1 month–11 months	54	38	0.95	0.28	3.18	.933
1 year–5 years	80	79	1.45	0.47	4.45	.518
6 years-10 years	34	31	1.37	0.42	4.41	.602
Above 10 years	11	8	Ref	-	-	-
Family history of diabetes or hypertension						
Yes	68	51	0.65	0.38	1.13	.126
No	111	105	Ref	-	-	-
Engaging in alcohol taking						
Yes	32	45	2.20	1.18	4.11	.013
No	147	111	Ref	-	-	-
Engaging in cigarette smoking						
Yes	7	2	0.28	0.05	1.67	.162
No	172	154	Ref	-	-	-
Do you participate in Physical exercises						
Yes	61	55	0.77	0.43	1.36	.358
No	118	101	Ref	-	-	-

## DISCUSSION

This study examined dietary knowledge and its associated factors among patients living with diabetes, hypertension, or both at two tertiary hospitals in Dodoma, Tanzania. The findings revealed that more than half of the participants had inadequate dietary knowledge, underscoring a substantial gap in nutritional understanding among individuals managing chronic NCDs. Several socio-demographic and behavioural factors including age, employment status, alcohol use, and type of diagnosis were significantly associated with dietary knowledge. These results highlight persistent challenges in nutrition education within NCD care and provide important insights for designing targeted interventions to improve self-management and health outcomes.

The study had more females than male respondents (62.1% versus 37.9%), this pattern was reported in previous studies conducted in Sudan, South Africa, Pakistan, Kenya, and Nepal.^26–29^ This consistency may be explained by the higher burden of obesity among women, which increases their susceptibility to diabetes and hypertension, as well as the tendency for women to be more proactive in seeking healthcare services.^30^ With respect to age, the majority of participants were older adults, particularly those aged above 60 years and those aged 45 to 60 years. Similar age distributions have been documented in previous studies conducted in the same countries, where advancing age was consistently identified as a major risk factor for both hypertension and diabetes.^26–29^ Although our findings show that these conditions affect all age categories, they remain more prevalent among older adults due to physiological changes associated with aging.

Most respondents were self-employed, and nearly three-quarters reported low income. This aligns with evidence from the 2018 Tanzania Nutrition and Health Survey and other related studies which showed that a large proportion of Tanzanians have low socioeconomic status and rely on monotonous diets dominated by carbohydrates, with limited practice of balanced meal planning.^31,32^ In this study, hypertension was the most common condition, followed by dual diagnosis (hypertension and diabetes), while diabetes alone was less frequent. These findings are consistent with previous research demonstrating that hypertension remains the most common cardiovascular condition in many populations.^33,34^ Additionally, most participants reported living with their condition for more than five years, reinforcing the chronic and lifelong nature of diabetes and hypertension.

The findings revealed that more than half of the participants had inadequate dietary knowledge, underscoring a major gap in patient understanding of nutritional requirements for effective NCD management. This prevalence is consistent with findings from studies conducted in Nepal, Saudi Arabia, Pakistan, and Singapore, which reported poor dietary knowledge among majority of their study participants,^28,29,32,35^ reflecting systemic challenges in patient education, limited access to nutrition counselling, and persistent socio-economic barriers. In contexts where health systems are overburdened and nutritionists are scarce, dietary counselling is often inconsistently provided, contributing to poor patient comprehension of healthy dietary practices.^36–38^ However, findings from studies conducted in Sudan, South Africa, and Uganda reported comparatively higher levels of dietary knowledge among respondents,^25–27^ which contrasts with the results of the present study. These differences may be attributed to variations in geographical context, accessibility and quality of dietary education programs, cultural norms, and differences in sample characteristics or study designs.

The finding that middle-aged adults had significantly higher odds of inadequate dietary knowledge compared with older adults may be explained by differences in health-seeking behaviours and exposure to health education. Older adults often live with chronic conditions for longer periods, resulting in more frequent clinic visits and greater interaction with healthcare providers, which increases their opportunities to receive dietary advice.^39^ Evidence also shows that older adults tend to engage more attentively with nutrition information and may be more receptive to lifestyle modification guidance.^40,41^ Furthermore, nutrition knowledge and competencies among older adults are often strengthened by repeated exposure to health education programs and community health initiatives.^39,42^ In contrast, middle-aged adults are typically more economically active, balancing work and family responsibilities, which limits their time for clinic attendance, participation in counselling sessions, or engagement with health information factors known to reduce opportunities for nutrition learning.^39,41^ These competing priorities may reduce their exposure to consistent dietary education, contributing to lower levels of dietary knowledge.

The association between unemployment and inadequate dietary knowledge highlights the role of socio-economic disadvantage in shaping health literacy. Unemployed individuals often experience financial barriers, limited access to health information, and challenges in prioritizing health over economic survival.^43,44^ Prior research has demonstrated that lower socioeconomic status is strongly linked to reduced health literacy and limited capacity to adopt recommended dietary practices.^45–47^ In addition, unemployment may restrict individuals’ ability to purchase diverse, healthier foods even when they possess some knowledge, thereby diminishing the perceived relevance of dietary education.^48,49^

The finding that participants who consumed alcohol had higher odds of inadequate dietary knowledge may indicate clustering of risk behaviors.^50^ Individuals engaging in alcohol use may have poorer adherence to medical advice and less engagement with lifestyle education.^51,52^ Alcohol consumption is also associated with reduced attention to long-term disease prevention, and it may compete with healthy dietary choices within limited household budgets.^53^ This finding corroborates previous studies suggesting that behavioural risk factors often coexist, creating cumulative barriers to healthy living.^54,55^

Interestingly, individuals diagnosed with hypertension only had significantly lower odds of inadequate dietary knowledge compared with those who had both diabetes and hypertension. Patients with dual diagnoses often face more complex treatment regimens and diverse dietary restrictions, which can complicate their understanding of appropriate dietary choices. They may also be overwhelmed by competing clinical advice, leading to confusion and suboptimal knowledge. This finding suggests that patients with multi-morbidity may require more tailored, simplified, and structured dietary counselling to overcome information overload and conflicting messages.

Overall, the study highlights significant gaps in dietary knowledge among NCD patients, with specific subgroups, such as middle-aged adults, unemployed individuals, and those who drink alcohol being particularly vulnerable. These gaps may hinder adherence to dietary recommendations and contribute to poor disease control, increased complications, and long-term health system burden.

### Implications for Practice and Policy

The findings highlight critical opportunities for strengthening clinical practice and public health approaches to NCD management in Tanzania. Routine and structured dietary counselling should be integrated into every NCD clinic visit, guided by standardized nutrition protocols and delivered by trained nutritionists or health educators. Targeted patient-centred interventions are needed for high-risk groups, particularly middle-aged adults, unemployed individuals, and those who consume alcohol, who demonstrated higher odds of inadequate dietary knowledge.

Empowering community health workers, establishing peer support groups, and implementing lifestyle modification classes may offer effective avenues for reaching these vulnerable populations. Additionally, improving the nutrition competencies of healthcare providers through continuous professional development would enhance the consistency and quality of dietary education delivered in clinical settings.

Beyond health facilities, broader public health and policy interventions are necessary to create environments that support healthy dietary choices. Community outreach programs, including mass media campaigns, digital health platforms, and community dialogues, can effectively disseminate practical, culturally relevant nutrition information to households and workplaces.

Addressing socio-economic barriers is equally critical; policies that increase access to affordable, healthy foods, such as fruit and vegetable subsidies or regulation of ultra-processed food prices, may help translate knowledge into sustainable practice among low-income populations. Moreover, individuals with multiple NCD conditions, such as those living with both hypertension and diabetes, require simplified, tailored dietary guidance to navigate complex recommendations. Developing clear “dualcondition diet guides” could reduce confusion, improve adherence, and ultimately lead to better disease control.

### Limitations

This study has several limitations. First, the cross-sectional design limits the ability to infer causal relationships between the identified factors and dietary knowledge. Second, the use of self-reported information may introduce recall or social desirability bias, especially for behavioural variables such as alcohol consumption. Third, the dietary knowledge instrument, although adapted from a validated tool, may not fully capture all culturally relevant aspects of dietary practices in Tanzania. Fourth, the study was conducted at two tertiary urban hospitals, which may limit generalizability to rural areas or lower-level health facilities where health literacy levels and access to information may differ. Lastly, the use of the mean score as the cut-off for adequate knowledge, although statistically acceptable, may complicate comparisons with other studies using standardized thresholds. Despite these limitations, the study provides robust and contextually relevant insights that can inform targeted interventions to enhance dietary knowledge and improve NCD management in Tanzania.

## CONCLUSION

This study revealed that more than half of patients living with diabetes, hypertension, or both had inadequate dietary knowledge, highlighting a significant gap in the nutritional understanding required for effective NCD self-management. Middle-aged adults, unemployed individuals, and those who consumed alcohol were more likely to exhibit inadequate dietary knowledge, suggesting that socio-economic and behavioural factors play a crucial role in shaping patients’ awareness of appropriate dietary practices. Conversely, patients diagnosed with hypertension alone were less likely to have inadequate knowledge compared with those with comorbid conditions. These findings underscore the need for targeted, context-specific dietary education interventions, particularly for socio-economically vulnerable groups, to strengthen NCD management and improve long-term health outcomes in Tanzania.
